# Functional imaging of interleukin 1 beta expression in inflammatory process using bioluminescence imaging in transgenic mice

**DOI:** 10.1186/1471-2172-9-49

**Published:** 2008-08-19

**Authors:** Limei Li, Zhaoliang Fei, Jianke Ren, Ruilin Sun, Zhihui Liu, Zhejin Sheng, Long Wang, Xia Sun, Jun Yu, Zhugang Wang, Jian Fei

**Affiliations:** 1 Laboratory of Molecular Cell Biology, Institute of Biochemistry and Cell biology, Shanghai Institutes for Biological Sciences, Chinese Academy of Sciences, Shanghai, PR China; 2 Shanghai Research Center for Model Organisms, Pu Dong, Shanghai, PR China; 3 School of Life Science and Technology, Tongji University, Shanghai, PR China; 4 Graduate School of Chinese Academy of Sciences, PR China; 5 Shanghai Genomics, Inc, Shanghai, PR China; 6 Shanghai Nan Fang Model Organism Research Center, No. 3577. Jinke Road, Pudong, Shanghai, People's Republic of PR China

## Abstract

**Background:**

Interleukin 1 beta (IL-1β) plays an important role in a number of chronic and acute inflammatory diseases. To understand the role of IL-1β in disease processes and develop an *in vivo *screening system for anti-inflammatory drugs, a transgenic mouse line was generated which incorporated the transgene firefly luciferase gene driven by a 4.5-kb fragment of the human IL-1β gene promoter. Luciferase gene expression was monitored in live mice under anesthesia using bioluminescence imaging in a number of inflammatory disease models.

**Results:**

In a LPS-induced sepsis model, dramatic increase in luciferase activity was observed in the mice. This transgene induction was time dependent and correlated with an increase of endogenous IL-1β mRNA and pro-IL-1β protein levels in the mice. In a zymosan-induced arthritis model and an oxazolone-induced skin hypersensitivity reaction model, luciferase expression was locally induced in the zymosan injected knee joint and in the ear with oxazolone application, respectively. Dexamethasone suppressed the expression of luciferase gene both in the acute sepsis model and in the acute arthritis model.

**Conclusion:**

Our data suggest that the transgenic mice model could be used to study transcriptional regulation of the IL-1β gene expression in the inflammatory process and evaluation the effect of anti-inflammatory drug *in vivo*.

## Background

The cytokine interleukin 1 beta (IL-1β) is a potent mediator in response to infection and injury [1]. It is produced mainly by blood monocytes, but also by macrophages, dendritic cells and a variety of other cells in the body [2,3]. A minute amount of IL-1β *in vivo *can evoke fever, hypotension, release of adrenocorticotrophic hormone and production of cytokines which in turn induce various inflammatory and immune responses.

Increased IL-1β production has been reported in patients with various infections, inflammation, trauma (surgery), ischemic diseases, tumors, intravascular coagulation, autoimmune disorders, UV radiation, graft-versus-host disease, transplant rejection, and in healthy subjects after strenuous exercise [4,5]. An increasing IL-1β production was observed in patients with Alzheimer's disease and a possible role for IL-1β in the release of the amyloid precursor protein was proposed [6]. Significant elevations of plasma IL-1β have been detected in healthy humans injected with lipopolysaccharide (LPS) and in patients with septic shock and burns [7]. Correlations have been found between plasma IL-1β levels and severity of acute attacks of rheumatoid arthritis, thermal burns, and mortality in septic shock [8]. Agents that reduce the production and activity of IL-1β are likely to have an impact on clinical applications. In fact, IL-1Ra, a blocker of IL-1β transduction, has been administered to patients with septic shock, rheumatoid arthritis, steroid resistant graft-versus-host disease, AML, CML and so on [8-11].

Development of a method to monitor IL-1β gene promoter activity *in vivo *will facilitate its use in the study of related diseases and preclinical evaluation of anti-inflammatory drugs. For this purpose, in this paper, we have established a transgenic mouse model using the human IL-1β gene promoter [12] to direct the expression of luciferase reporter gene. When combining with the approach of "biophotonic" imaging using a highly light-sensitive camera system [13-15], this model allows us to non-invasively study the transcriptional activity of IL-1β gene promoter in real time. Our data indicate that human IL-1β gene promoter functions in transgenic mice and this model can be used to study transcriptional regulation of the IL-1β gene expression in the inflammatory process and evaluate the effects of anti-inflammatory agents on IL-1β gene induction *in vivo*.

## Results

### Founder screening and molecular characterization

**Figure 1:**
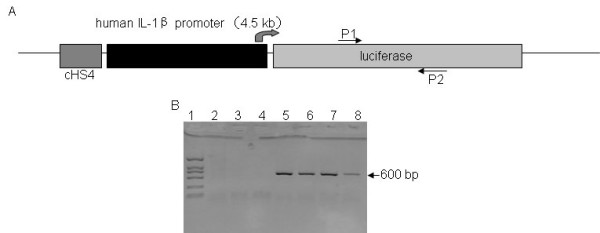
**Schematic diagram of IL-1β-luc reporter system used for microinjection**. (A) The cHS4I-hIL-1βP-Luc transgene was constructed by inserting a 4.5-kb 5' flanking promoter region of the human IL-1β gene in front of firefly luciferase cDNA. (B) Genotyping by PCR yielded a 600-bp fragment. PCR products were run on a 1% agarose gel. Lane 1 was a DL 2,000 DNA ladder, lane 2 was the product from a wild-type CBA control, lane 3 was the product from a wild-type C57BL/6 control, lane 4 was the buffer for dissolving the genomic DNA, and lanes 5–8 were samples from cHS4I-hIL-1βP-Luc heterozygous transgenic mice. P1: forward-luc primer, P2: reverse-luc primer.

The plasmid used for construction of transgenic mice was illustrated in Fig. 1A. Transgenic founders is identified by PCR detection of luciferase gene (using the primer pair marked in Fig. 1A) in tail-clip DNA (Fig. 1B). Four founder mice were obtained and crossed to BALB/c mice for five generations to generate progeny for further experiments.

### Induction of luciferase expression in cHS4I-hIL-1βP-Luc transgenic mice by LPS

**Figure 2:**
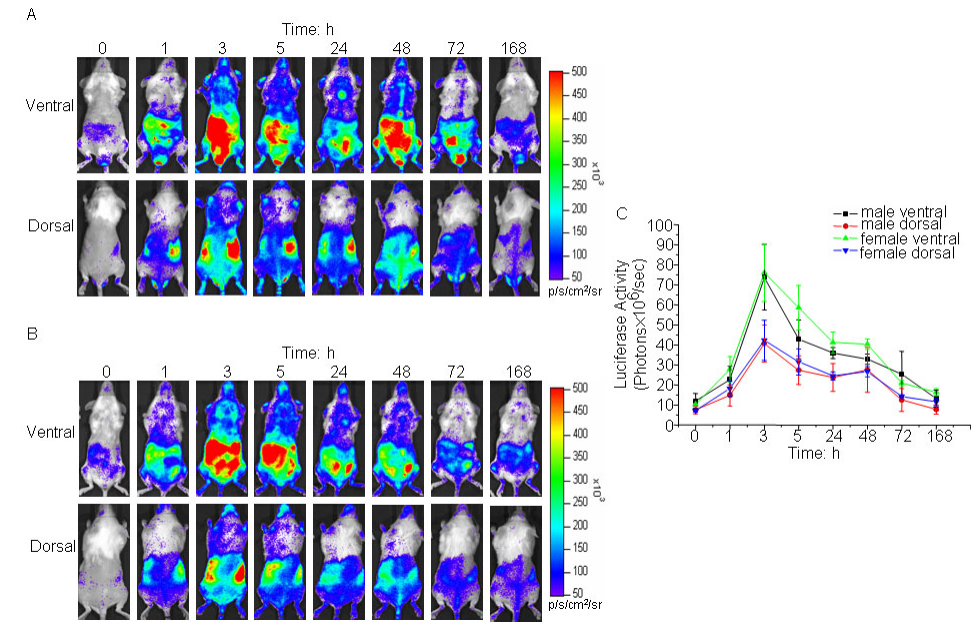
**Induction of hIL-1βP-driven luciferase expression in IL-1β-luc transgenic mice by LPS**. Dorsal and ventral views of a representative mouse are depicted for male (A) and female (B) mice. The color overlay on the images represents the photons/sec/cm^2^/steradian (p/s/cm^2^/sr), as indicated by the color scale next to the images. (C) Quantification of luciferase activity (photons/sec) of the whole body with the LivingImage software. Data presented are group means of 3 mice ± standard errors.

The progenies of cHS4I-hIL-1βP-Luc transgenic founders were screened for luciferase expression in response to LPS as described in materials and methods. All transgenic lines showed robust inducible luciferase activity in the whole body after LPS treatment while injection with saline did not induce luciferase expression. One line named BALB/cTg(cHS4I-hIL-1βP-Luc)Xen had the highest LPS-induced luciferase expression (Fig. 2) and was selected for further studies. In these mice, luciferase activity was detectable 1 h (*n *= 3/group, *p *> 0.05 in males compared with the baseline level, *p *< 0.01 in females compared with the baseline level) after LPS treatment all over the body and relatively higher expression levels were seen at the position of liver, intestine and lungs. The expression signal peaked at 3 h (*n *= 3/group, *p *< 0.001 in males and females compared with the baseline level, respectively) after treatment and then gradually declined; by 168 h (*n *= 3/group, *p *> 0.05 in males compared with the baseline level, *p *< 0.01 in females compared with the baseline level) the signal had returned to the baseline level (Fig. 2A, B). As quantified by the LivingImage software, both male and female mice showed significant LPS-induced luciferase signal above the baseline level from 1 to 72 h after treatment, and the kinetics and magnitude of the signals were similar in the two sexes (Fig. 2C). At the peak, the luciferase signal was induced by 7–8 fold in both sexes. In another experiment, no significant difference in LPS-induced luciferase expression was found among mice aged from 2 to 7 months (data not shown).

### Expression of luciferase was induced in parallel with mouse endogenous IL-1β in multiple tissues after LPS treatment

**Figure 3:**
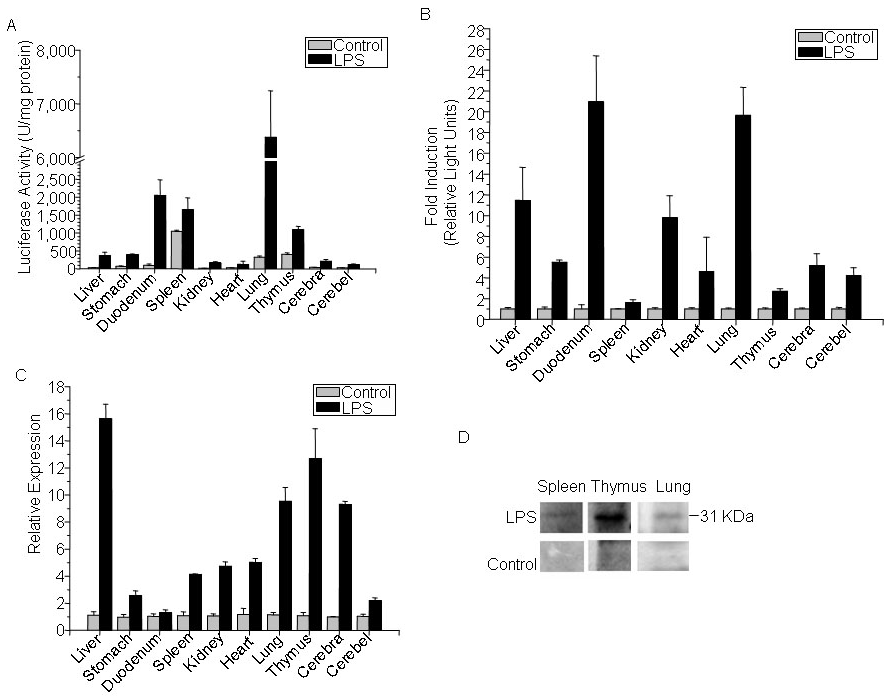
**hIL-1βP-driven luciferase expression is induced in multiple tissues of IL-1β-luc transgenic mice after LPS treatment**. (A) Female cHS4I-hIL-1βP-Luc mice (n = 3) were treated with LPS (3 mg/kg). At 5 h after LPS injection, mice were sacrificed and selected organs were rapidly harvested and homogenized for luciferase activity measurement with a luminometer. Saline-treated mice were used as controls. (B) Fold induction of the luciferase expression in the selected organs. Degree of change in gene expression is based on the expression level of the saline-treated mice. All values were expressed as mean ± SE. (C) Quantification-RT-PCR analysis for IL-1β gene expression in female mice. The mRNA levels of IL-1β gene expression in the selected organs of LPS-treated mice were quantified using real-time RT-PCR with Evagreen detection at 5 h after treatment. Degree of change in gene expression is based on the expression level of the saline-treated mice. All values were expressed as mean ± SE (n = 3/group). (D) Western blot for the presence of pro-IL-1β protein in the spleen, thymus and lung at 5 h following treatment with saline or LPS.

To confirm the *in vivo *activity data observed by live imaging, in another set of experiments, luciferase activity was detected *ex vivo *in dissected organs of cHS4I-hIL-1βP-Luc female mice at 5 h after LPS injection (Fig. 3A). The luciferase activity was significantly higher in the liver, lungs, duodenum, kidneys, brain, and heart. As compared with the saline treated mice, LPS treatment induced the luciferase activity by 11-fold in the liver, 19.6-fold in the lungs, 21-fold in the duodenum, 10-fold in the kidneys, 5-fold in the brain, 4.5-fold in the heart, 5.5-fold in the stomach, 2.7-fold in the thymus, and 1.5-fold in the spleen (Fig. 3B), which was consistent with the *in vivo *results at 5 h in Fig. 2B.

The LPS-induced expression of mouse endogenous IL-1β mRNA was evaluated by real-time RT-PCR (Fig. 3C). In a large degree in parallel with that of the induced luciferase expression, LPS treatment increased endogenous IL-1β mRNA expression by 15.6-fold in the liver, 9.5-fold in the lungs, 4.7-fold in the kidneys, 9-fold in the brain, 5-fold in the heart, 2.5-fold in the stomach, 12.7-fold in the thymus, 4.2-fold in the spleen, and 1.3-fold in the duodenum. Western Blot analysis showed that LPS significantly induced the expression of pro-IL-1β protein in lung, spleen, and thymus of mice (Fig. 3D).

### Luciferase expression in cHS4I-hIL-1βP-Luc transgenic mice was inducible during acute arthritis

**Figure 4:**
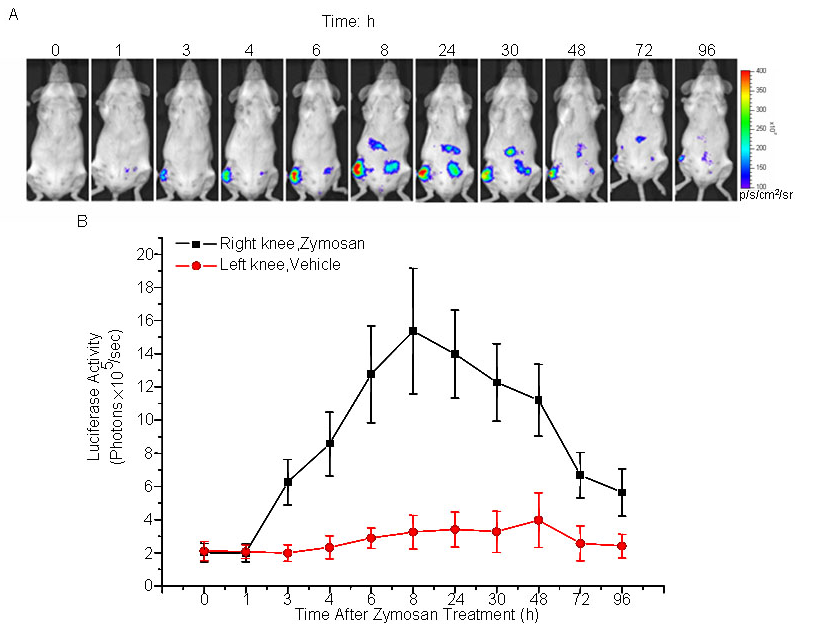
**Monitoring hIL-1βP-driven luciferase expression in IL-1β-luc transgenic mice during zymosan-induced acute arthritis**. (A) Female cHS4I-hIL-1βP-Luc mice (n = 3/group). were injected with zymosan (300 μg/knee) into the right rear knee joint, and vehicle (5% glucose in PBS) into the left rear knee joint. The mice were imaged at 0, 1, 3, 4, 6, 8, 24, 30, 48, 72, and 96 h following injection. The color overlay on the images represents the photons/sec/cm^2^/steradian (p/s/cm^2^/sr), as indicated by the color scale next to the images. (B) Quantification of luciferase activity (photons/sec) of the knee region by the LivingImage software. Data presented were mean ± SE.

The inducibility of IL-1β gene expression during acute inflammatory arthritis was studied using the cHS4I-hIL-1βP-Luc transgenic mouse model. Intra-articular injection of zymosan into the right knee joint of cHS4I-hIL-1βP-Luc mice caused a local induction of luciferase signal which was detectable at 2 h, peaked at 8 h, started to decline at 24 h and was still detectable at 96 h after the local treatment (Fig. 4A, B). The luciferase signal at the zymosan-injected right knee joints were 3.1-, 7.6-, 6.9-, and 2.8-fold of the base level at 3, 8, 24, and 96 h, respectively (Fig. 4B). The saline injected left knees showed slight increase in luciferase signal, possibly caused by a systemic response to zymosan administration and/or the lesion at the injection site. The induction of luciferase signal correlated with an increase of knee joint volume, as measured across lateral/medial axis and the anterior/posterior axis of the knee joints (results not shown).

### Luciferase expression was inducible during CHS reaction

**Figure 5:**
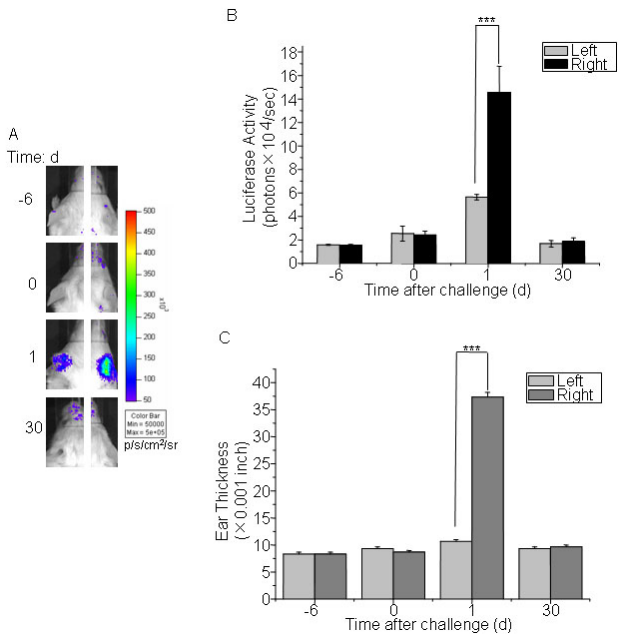
**hIL-1βP-driven luciferase expression in IL-1β-luc transgenic mice in an oxazolone-CHS model**. (A) Female cHS4I-hIL-1βP-Luc mice (n = 3) are sensitized with oxazolone on day -6. On day 0, the right ears were challenged with oxazolone and the left ears were treated with vehicle. Imaging analysis was performed on day – 6, 0, 1, and 30. (B) Quantification of luciferase expression (photons/sec) in all the ears with LivingImage software. Data were presented as mean ± SE. (C) Ear thickness was measured on day – 6, 0, 1, and 30 with a micrometer. Data represent mean ± SE. *** indicate significant differences at p < 0.001. p/s/cm^2^/sr: photons/sec/cm^2^/steradian.

In a third model of inflammatory disease, cHS4I-hIL-1βP-Luc female mice were sensitized with oxazolone and topically challenged at the right ear with oxazolone 6 days later. Oxazolone challenge induced luciferase expression in the right ears at the level of 2.6 fold of the base level at day 1 after challenge (Fig. 5B). A weak induction of luciferase expression was also observed in the vehicle injected left ear. Induction of luciferase expression correlated with increase in ear thickness (Fig. 5C).

### Dexamethasone inhibited the induction of luciferase expression

**Figure 6:**
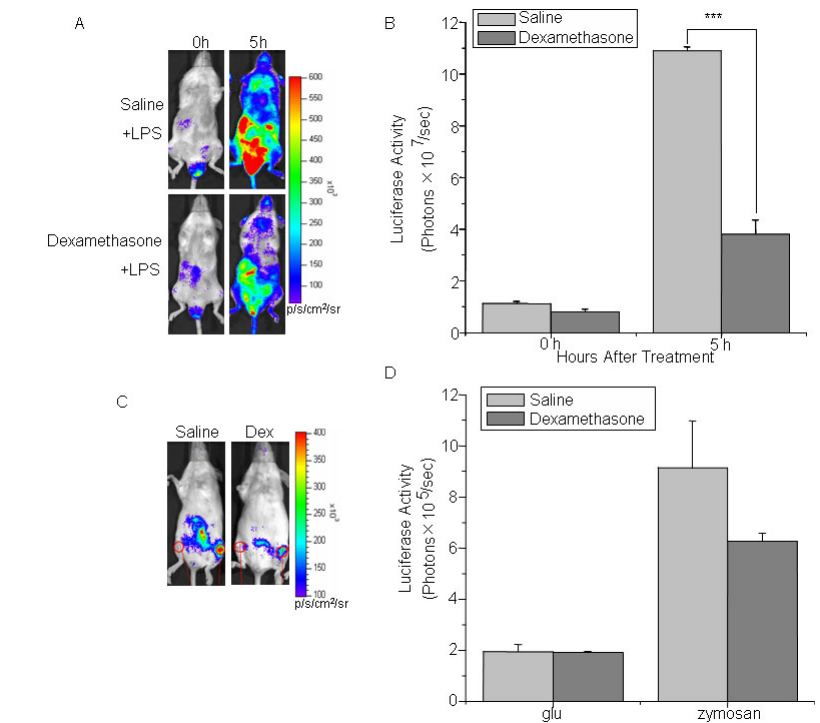
**Dexamethasone inhibits hIL-1βP-driven luciferase expression in IL-1β-luc transgenic mice in the LPS and zymosan induced inflammation models**. (A) Dexamethasone inhibits LPS-induced hIL-1βP-driven luciferase expression. Male cHS4I-hIL-1βP-Luc mice (n = 3/group) were cotreated with vehicle or dexamethasone (3 mg/kg) during the LPS (3 mg/kg) injection. All the mice were imaged at 0 and 5 h after LPS injection. (B) Quantification of luciferase activity (photons/sec) with the LivingImage software. Data presented were mean ± SE. (C) Dexamethasone inhibits hIL-1βP-driven luciferase expression during zymosan induced acute arthritis. Female cHS4I-hIL-1βP-Luc mice (n = 3/group) were cotreated with vehicle or dexamethasone (50 μg/mouse) during the administration of zymosan (300 μg/mouse). All the mice were imaged at 0 and 4 h after zymosan injection. (D) Quantification of luciferase activity (photons/sec) of the articular region of the acute arthritis mice with the LivingImage software. Data presented were mean ± SE. *** indicate significant differences at p < 0.001. p/s/cm^2^/sr: photons/sec/cm^2^/steradian.

Dexamethasone, a synthetic glucocorticoid, has been well characterized for its ability to inhibit IL-1β production. A major mechanism is that dexamethasone inhibits IκB degradation and therefore suppresses IL-1β gene transcription promoted by NF-κB signaling pathway [16,17]. Male cHS4I-hIL-1βP-Luc mice were cotreated with LPS and dexamethasone (3 mg/kg i.p.) or LPS and saline. The dexamethasone cotreated mice had 51.5% lower induced luciferase expression as compared with the saline cotreated mice (Fig. 6A, B). Similarly, dexamethasone inhibited the induced luciferase expression in the zymosan induced acute arthritis model (Fig. 6C, D).

## Discussion

We have generated a cHS4I-hIL-1βP-Luc transgenic mouse model for monitoring hIL-1β gene transcriptional activity during inflammation. Using an *in vivo *bioluminescence imaging system, we demonstrated that luciferase signals in the cHS4I-hIL-1βP-Luc transgenic mice were dramatically induced in the whole body (especially in liver, duodenum, lung, kidneys, stomach, brain, and thymus) following treatment with LPS. Direct quantification of luciferase signals using the LivingImage software yielded data comparable to those obtained by the conventional luminometer assay performed on excised and homogenized tissues. In an arthritis and a CHS reaction model, luciferase signals were induced locally in the treated area. Dexamethasone, a well known blocker of IL-1β production [16-18], suppressed the induced expression of luciferase in transgenic mice in these inflammation models. These data indicate that cHS4I-hIL-1βP-Luc mice are useful as a sensitive and convenient model for monitoring IL-1β gene expression during the disease process in a broad range of inflammatory conditions and for evaluating the effects of anti-inflammatory drugs.

Conventional methods for monitoring IL-1β gene expression rely mostly on either measuring circulating levels of IL-1β in the serum or mRNA expression in tissues. Compared with these methods, the approach reported in this study is convenient and sensitive, and more importantly, with this approach the kinetics as well as the anatomical location of IL-1β gene expression can be conveniently studied and use a minimal number of animals. This is extraordinarily useful in monitoring both systematic and local inflammation, such as sepsis, arthritis, and chemical-induced skin toxicity.

The pattern of LPS-induced luciferase expression in transgenic mice was generally in agreement with the endogenous murine IL-1β mRNA expression. However, the fold of increase did not match exactly between luciferase activity and endogenous IL-1β mRNA. This might be explained by the facts that the promoter for the luciferase is from human and IL-1β mRNA level and protein level are often not in linear correlation. In addition, translational regulation of IL-1β mRNA and luciferase mRNA, as well as posttranslational regulation of IL-1β protein and luciferase protein, may also be different. So it should be note that the signal of luciferase in the reporter mice may be not consist with the IL-1β protein level since the regulation of IL-1β protein translational and posttranslational process is rather complex in vivo[19]. However, these differences do not affect the application of our transgenic model in inflammation research to monitor the mRNA level of IL-1β. In the cHS4I-hIL-1βP-Luc transgenic mice described here, there were no clear gender differences in both baseline of luciferase signals and response to LPS where nearly identical kinetics and signal intensity were observed. This result is in agreement with the results reported previously [20].

IL-1β have been found in the synovial fluid recovered from inflamed joints of rheumatoid arthritis patients [21]. We clearly demonstrated that local administration of zymosan into knee joints triggered induction of luciferase expression in the joints, which correlated with the increases in both IL-1β mRNA and protein in zymosan-treated knee joint tissue reported earlier [22]. It is believed that locally synthesized IL-1β by synovial cells in inflamed joints plays a key role in the pathogenesis of rheumatoid arthritis, and IL-1β can be used as a marker for evaluating the efficacy of therapeutics for rheumatoid arthritis.

Luciferase expression was also clearly induced in the ear of the transgenic mice in a CHS model. Contact hypersensitivity is a two-phase process. The induction phase begins on initial epicutaneous application of a hapten (low-molecular-weight chemical), resulting in activation and rapid lymphocyte proliferation. The challenge or elicitation phase occurs when sensitized individuals are re-exposed to the sensitizing antigen. This local CHS reaction is characterized by infiltrating T lymphocytes, macrophages, and neutrophils. It has been reported that mice with oxazolone treatment in the ear showed ear swelling and increased expression levels of IL-1β, IL-4, IL-18 and GM-CSF [23,24]. In line with these reports, we showed that luciferase expression was induced in the ear of the transgenic mice at day 1 after oxazolone challenge and the expression level correlated with the increase in ear thickness.

The regulatory region for human IL-1β gene expression was found to be distributed over several thousand basepairs upstream and a few basepairs downstream from the transcriptional start site [25]. There are two independent enhancer regions, -2782 to -2729 and -2896 to -2846 that appear to act cooperatively [26]. The latter contains a cAMP response element, whereas the former is a composite cAMP response element (NFIL-6) that is responsive to LPS. The 80-bp fragment (-2800 to -2720) is required for transcription and contains, in addition to a cAMP response element, an NF-κB-like binding site. Activating protein-l (AP-l) sites also participate in endotoxin-induced IL-1β gene expression. Proximal promoter elements have also been identified between -131 and +14 [26]. Sequences in this region contain binding sites for the novel nuclear factor NFβA [27], which appears to be similar to nuclear factors termed NFβl and NFβ2 [26]. This proximal promoter is required for maximal IL-1β gene expression. The nucleotide binding sequences of NFβA are found to be identical to those of the transcription factor Spi-1/PU.1 [28,29], a well-established NF in cells of myeloid and monocyte lineage [30]. The requirement for Spi-1/PU.1 for IL-1β gene expression imparts its tissue specificity. Human blood monocytes, which constitutively express Spi-l/PU.1, are exquisitely sensitive to LPS inducible IL-1β gene expression. In our case, the promoter region used for driving luciferase expression in the transgenic mice is 4.5-kb long which contains all the cis-elements discussed above. The chicken HS4 insulator (cHS4I) upstream of the hIL-1βP may eliminate the transgenic chromosomal insertion site effect on transgene expression[31]. Therefore, the expression of the luciferase gene in the transgenic mice was expected to reflect the natural pattern of IL-1β gene expression.

Bioluminescence imaging techniques have been used to study the expression of several inflammatory factors in reporter mice, such as NF-κB-binding cis elements[13,15], the inducible NO synthase gene(iNOS)[32], the vascular endothelial growth factor-2 (VEGFR2)[33]. These reporter systems can be successfully used to monitor and quantify different inflammatory processes in vivo. Together with our model in this paper, we believe these reporter mice systems will facilitate us a better understanding of the molecular mechanism of different diseases.

## Conclusion

The cHS4I-hIL-1βP-Luc transgenic mouse, with its IL-1β promoter driven luciferase expression, is able to provide information about anatomical sites of IL-1β expression in an inflammatory process. The induction was observed in the whole body (especial in liver, lungs, and duodenum) in a LPS-sepsis model, and was restricted to the treated joints and ears in a zymosan-arthritis model and in an oxazolone-CHS model, respectively. Treatment with dexamethasone, a proven IL-1β expression blocker, significantly suppressed LPS- or zymosan-induced IL-1β promoter-driven luciferase expression. As IL-1β induction is featured in the pathogenesis of a number of acute and chronic inflammatory diseases, the cHS4I-hIL-1βP-Luc mouse is a useful tool not only for tracking various inflammatory processes *in vivo*, but also for testing the efficacies of therapeutic compounds that are targeted to inflammatory diseases, especially those that involve induction of IL-1β expression.

## Methods

### Reagents

Bacterial LPS (from Escherichia Coli Serotype 0127:B8), zymosan A (a cell wall preparation from Saccharomyces cerevisiae), dexamethasone and oxazolone were purchased from Sigma-Aldrich (St. Louis, MO, USA). Luciferin (Biosyth, Basel, Switzerland) was dissolved in PBS at 15 mg/ml and stored at -20°C.

### Generation of cHS4I-hIL-1βP-Luc transgenic mice

A 4.5-kb *Kpn *I-*Bgl *II fragment of human IL-1β gene promoter (hIL-1βP) was isolated from human genomic DNA by PCR amplification according to the literature [12]. The promoter fragment was verified by DNA sequencing and cloned into the polylinker sites *Kpn *I and *Bgl *II of pGL3-Basic (Promega) to direct the expression of luciferase reporter gene (hIL-1βP-Luc). The resulting plasmid was named as pGL3-hIL-1βP-Luc. In order to eliminate the transgenic chromosomal insertion site effect on transgene expression[31], the *Not *I-*Kpn *I fragment of the chicken HS4 insulator (cHS4I) was cloned into the *Not *I and *Kpn *I sites upstream to hIL-1βP. Then, the constructed cHS4I-hIL-1βP-Luc cassette was cut and subcloned into the *Apa I *and *Sal *I sites of plasmid pGFP-1. The cHS4I-hIL-1βP-Luc transgenic cassette (Fig. 1A) was cut out by *Apa *I and *Mlu *I, and used to generate transgenic mice in the C57XCBA background by standard microinjection techniques. The transgenic mice were bred to BALB/c for 5 generations before testing. All animals were housed in a specific pathogen-free environment in accordance with institutional guidelines for animal care. Animal handling was performed in accordance with institutional guidelines and approved by the local institutional animal care and use committee.

### Genotyping of cHS4I-hIL-1βP-Luc transgene in mice

Transgenic founders and their offspring were identified by PCR using the forward-luc (5' TTCCGCCCTTCTTGGCCTTTATGA 3') and reverse-luc (5' CAGCTATTCTGATTACACCCGAGG 3') primers specific for luciferase gene.

### *In vivo *imaging

*In vivo *bioluminescent imaging was performed using an IVIS imaging system (Xenogen, Alameda, CA) as previously described. 150 ul sodium salt luciferin (dissolved in PBS) was injected into the intraperitoneal cavity at a dose of 150 mg/ml. Mice were anesthetized with isoflurane/oxygen and placed on the imaging stage. 12 min after luciferin injection, mice were imaged for 1 to 5 min. Photons emitted from specific regions were quantified using a LivingImage software (Xenogen). *In vivo *luciferase activity was presented in photons emitted per second.

### Functional screening of cHS4I-hIL-1βP-Luc transgenic lines

For primary functional screening of transgenic lines, the luciferase gene PCR positive mice were imaged for expression of the luciferase transgene without any treatment or at 5 h after LPS injection at the does of 3.0 mg/kg. The criteria used for screening available transgenic lines were: (1) lower basal luciferase expression in normal status and (2) higher induced luciferase expression in whole body after LPS injection.

### Acute septic shock model by intraperitoneal LPS injection

The acute septic shock model was produced by i.p. injection of LPS (3.0 mg/kg) into cHS4I-hIL-1βP-Luc transgenic mice at the age of 2–3 mo. Control mice were injected with saline. At selected time points after the treatment, mice were i.p. injected with luciferin and imaged 12 min later with the IVIS imaging system described above. To test the effect of dexamethasone on LPS-triggered luciferase expression, male mice were cotreated with dexamethasone and LPS, and the control mice were injected with saline and LPS. The luciferase signal was monitored through imaging.

### Acute arthritis model by intra-articular administration of zymosan

Zymosan A was suspended in sterile saline containing 5% glucose at a concentration of 30 mg/ml. Female cHS4I-hIL-1βP-Luc mice at the age of 2–3 mo were anesthetized with isoflurane. Then the hind legs of mice were shaved and the skin was sterilized with 70% ethanol. The right knee tendon was exposed and 10 μl of zymosan A suspension was intra-articularly injected through the tendon with a 25-gauge needle. The left rear knees of the same mice were intra-articularly injected with 5% glucose in saline (sham control). Mice were imaged at selected time points after the injection. Separately, mice were pretreated with dexamethasone (50 μg/mouse i.p.) half hour before the zymosan administration, and the luciferase signal was monitored through imaging.

### Induction of contact hypersensitivity (CHS) reaction

Female cHS4I-hIL-1βP-Luc mice, 3–6 mo of age, were shaved in the thorax area and treated topically with 50 μl of 2% oxazolone solution (in acetone/olive oil (4/1, vol/vol)). Six days after sensitization, the right ears were challenged by topical application of 10 μl of 1% oxazolone solution, and the left ears were treated with vehicle (acetone/olive oil, 4:1 vol/vol) alone. The extent of inflammation was assessed by measuring ear thickness with a micrometer. Bioluminescent images were collected on the day of sensitization (day -6), the day of challenge (day 0), and at day 1 and 30 after treatment.

### Luciferase activity assay *in vitro*

Luciferase activity was assayed using the Luciferase Assay System (Promega) on a Luminometer (Lumat LB9507, EG&G, Berthold, Germany).

### Total RNA isolation

Female cHS4I-hIL-1βP-Luc mice, 3–6 mo of age, were treated with i.p. injection of LPS (3.0 mg/kg). Control mice were injected with saline. After 5 h, total RNA was isolated from selected mouse tissues using TRIZOL (Invitrogen, Paisley, Scotland, UK) according to the manufacturer's instruction and the extracted RNAs were kept at -80°C prior to use.

### Real-time reverse transcription-polymerase chain reaction (real time RT-PCR)

Two μg of the RNA samples isolated from the LPS-treated female cHS4I-hIL-1βP-Luc mice was reverse-transcribed into cDNA using M-MLV reverse transcriptase (Promega) in a final reaction volume of 25 μl. For real time PCR amplification, 0.5 μl of cDNA per reaction and 0.2 μM primer sets were used. Reaction conditions were as follows: 94°C/4 min; 51 cycles of 94°C/30 s, 60°C/30 s, 72°C/30 s; and 72°C/10 min. Evagreen used for detecting PCR products was purchased from Molecular Probes (Gentaurer, Brussels, Belgium). Amplification and detection of Evagreen were performed with RG-3000 Real Time Thermal Cycler (Corbett. Research, Sydney, Australia). The expression of the mRNA for the murine β-actin gene was used as a reference to normalize expression levels. All data were expressed relative to β-actin to compensate for any difference in sample loading, and all experiments were performed in triplicates. Data were obtained as threshold cycle (Ct) values (PCR cycle numbers at which fluorescence was threshold) using Rotor-Gene Real-Time Analysis Software 6.0 and used to calculate ΔCT values (ΔCT is the Ct of the target gene subtracted from the Ct of the housekeeping gene). Fold changes relative to saline controls were determined by the 2-ΔΔCT method.

Primers for real time PCR were synthesized as the sequences listed below (Sangon, Shanghai, China). The forward murine IL-1β primer used was 5' AAGGAGAACCAAGCAACGACAAAA 3', the reverse murine IL-1β primer used was 5' TGGGGAACTCTGCAGACTCAAACT 3'. The forward murine β-actin primer used was 5' CCTGTATGCCTCTGGTCGTA 3', the reverse murine β-actin primer used was 5' CCATCTCCTGCTCGAAGTCT 3'.

### Western blot analysis of mIL-1β and luciferase activity assay in tissues

Mouse tissues were removed and homogenized in 3 volumes of phosphate buffered saline (PBS) containing a protease inhibitor cocktail (Roche Applied Science, Indianapolis, IN, USA) and lysed with passive lysis buffer (Promega). After centrifugation at 14,000 rpm for 10 min at 4°C, the supernatant was collected. Luciferase activity was measured using a luminometer and the Luciferase Assay System (Promega). Protein concentration in lysate was estimated by Bradford reagent (Sangon, Shanghai, China). For Western blot analysis, equal amounts of proteins (120 μg) were loaded onto SDS-PAGE gels. After transfer, the membrane was probed with a specific anti-mIL-1β rabbit polyclonal Ab (Chemicon International, Temecula, CA, USA). The blots were developed using the ECL Plus Western Blotting Detection System (Amersham Biosciences, Piscataway, NJ, USA).

### Statistics

All data are expressed as means ± SE. The data were analyzed by one-way ANOVA. A P value of less than 0.05 was considered significant.

## Authors' contributions

LL and JF designed and analyzed all the experiments, and wrote the manuscript. LL carried out the molecular genetic studies and the immunoassays, participated in the sequence alignment and the design of the study, and performed the statistical analysis. LW and XS participated in transgenic research. ZF, JR, RS, ZL, ZS, JY and ZW participated in its design and coordination. All authors read and approved the final manuscript.
